# Recommendations for patient involvement in health technology assessment in Central and Eastern European countries

**DOI:** 10.3389/fpubh.2023.1176200

**Published:** 2023-07-03

**Authors:** Ivett Jakab, Maria Dimitrova, François Houÿez, Tamás Bereczky, Miroslava Fövényes, Zorana Maravic, Ivica Belina, Christian Andriciuc, Krisztina Tóth, Oresta Piniazhko, Rok Hren, Iñaki Gutierrez-Ibarluzea, Marcin Czech, Tomas Tesar, Maciej Niewada, László Lorenzovici, Maria Kamusheva, Manoela Manova, Alexandra Savova, Zornitsa Mitkova, Konstantin Tachkov, Bertalan Németh, Zsuzsanna Ida Petykó, Dalia Dawoud, Diana Delnoij, Saskia Knies, Wim Goettsch, Zoltán Kaló

**Affiliations:** ^1^Syreon Research Institute, Budapest, Hungary; ^2^Patient advocate, Budapest, Hungary; ^3^Faculty of Pharmacy, Medical University of Sofia, Sofia, Bulgaria; ^4^European Organisation for Rare Diseases (EURORDIS), Paris, France; ^5^Patvocates GmbH, Riemerling, Germany; ^6^Lymfoma Slovensko, Bratislava, Slovakia; ^7^Digestive Cancers Europe, Brussels, Belgium; ^8^Coalition of Associations in Healthcare, Zagreb, Croatia; ^9^Romanian Federation of Diabetes Associations, Cluj Napoca, Romania; ^10^Bridge of Health Alliance against Breast Cancer Association, Budapest, Hungary; ^11^Health Technology Assessment Department of State Expert Centre, Ministry of Health of Ukraine, Kyiv, Ukraine; ^12^Faculty of Mathematics and Physics, University of Ljubljana, Ljubljana, Slovenia; ^13^Basque Foundation for Health Innovation and Research, Landa, Spain; ^14^Head of Pharmacoeconomic Department, Institute of Mother and Child, Warsaw, Poland; ^15^Department of Organisation and Management in Pharmacy, Faculty of Pharmacy, Comenius University, Bratislava, Slovakia; ^16^Department of Experimental and Clinical Pharmacology, Medical University of Warsaw, Warsaw, Poland; ^17^Syreon Research Romania, Tirgu Mures, Romania; ^18^Department of Doctoral School of Medicine and Pharmacy, George Emil Palade University of Medicine, Pharmacy, Science, and Technology, Targu Mures, Romania; ^19^National Council on Prices and Reimbursement of Medicines, Sofia, Bulgaria; ^20^Centre for Health Technology Assessment, Semmelweis University, Budapest, Hungary; ^21^Science, Policy and Research Programme, National Institute for Health and Care Excellence, London, United Kingdom; ^22^National Health Care Institute (ZIN), Diemen, Netherlands; ^23^WHO Collaborating Centre for Pharmaceutical Policy and Regulation, Division of Pharmacoepidemiology and Clinical Pharmacology, Utrecht University, Utrecht, Netherlands

**Keywords:** patient involvement, health technology assessment, reimbursement, decision-making, Central and Eastern Europe countries, stakeholder perspectives, barrier prioritisation, recommendations

## Abstract

**Introduction:**

Meaningful patient involvement in health technology assessment (HTA) is essential in ensuring that the interests of the affected patient population, their families, and the general public are accurately reflected in coverage and reimbursement decisions. Central and Eastern European (CEE) countries are generally at less advanced stages of implementing HTA, which is particularly true for patient involvement activities. As part of the Horizon2020 HTx project, this research aimed to form recommendations for critical barriers to patient involvement in HTA in CEE countries.

**Methods:**

Built on previous research findings on potential barriers, a prioritisation survey was conducted online with CEE stakeholders. Recommendations for prioritised barriers were formed through a face-to-face workshop by CEE stakeholders and HTx experts.

**Results:**

A total of 105 stakeholders from 13 CEE countries completed the prioritisation survey and identified 12 of the 22 potential barriers as highly important. The workshop had 36 participants representing 9 CEE countries, and 5 Western European countries coming together to discuss solutions in order to form recommendations based on best practices, real-life experience, and transferability aspects. Stakeholder groups involved in both phases included HTA organisation representatives, payers, patients, caregivers, patient organisation representatives, patient experts, health care providers, academic and non-academic researchers, health care consultants and health technology manufacturers/providers. As a result, 12 recommendations were formed specified to the CEE region’s context, but potentially useful for a broader geographic audience.

**Conclusion:**

In this paper, we present 12 recommendations for meaningful, systematic, and sustainable patient involvement in HTA in CEE countries. Our hope is that engaging more than a hundred CEE stakeholders in the study helped to spread awareness of the importance and potential of patient involvement and that the resulting recommendations provide tangible steps for the way forward. Future studies shall focus on country-specific case studies of the implemented recommendations.

## Introduction

Compared to other regions of Europe, Central and Eastern European (CEE) countries generally have fewer public resources for health care and people living there are in a worse health status; therefore, they have an even greater need to reduce the opportunity cost of policy decisions by considering the best available scientific evidence (1). Health technology assessment (HTA) is a multidisciplinary process that uses explicit methods to determine the value of a health technology at different points in its lifecycle (2). The purpose is to inform decision-making in order to promote an equitable, efficient, and high-quality health system (2). Meaningful patient and public involvement in HTA is essential in ensuring that the interests of the affected patient population, their families and the general public can be accurately reflected in coverage and reimbursement decisions (3).

The methodology and level of patient involvement in HTA is determined at the national and regional levels, in line with the entire process of HTA (4). There are European-level initiatives on HTA such as the European network for Health Technology Assessment (EUnetHTA);^1^ and the new harmonized regulation on HTA^2^ now adopted in the European Union (EU) with joint assessments starting from January 2025. However, EUnetHTA only covered partial HTA of selected technologies, and the EU regulation will only cover the relative effectiveness assessment and not other parts of HTA. For instance, cost-effectiveness will continue to be assessed on the national level. Additionally, national and regional HTA bodies will remain to have a major role in coordinating, assessing and co-assessing joint HTA assessments. These HTA bodies will have a legal obligation, as per Article 8 of the HTA Regulation, to “take into account input received from patients, clinical experts and other relevant experts” (see text footnote 2). Thereby, there is still and will remain, a need for individual EU countries to conduct HTA with proper patient involvement, and a continued need of similar efforts for countries outside the EU.

With some exceptions, CEE countries are generally at less advanced stages of implementing HTA (1), which is particularly true for patient involvement in HTA (5). Patient involvement practices are limited in CEE countries lacking clear methodologies or regulatory mechanisms to guide patient involvement in the HTA process (5). This raises the question of transferability of practices used in other countries and calls for the development of new CEE-specific recommendations.

This research was conducted as part of the HTx Horizon 2020 project.^3^ The main aim of HTx is to create a framework for the next generation Health Technology Assessment to support patient-centred, societally oriented, real-time decision-making on access to and reimbursement for health technologies throughout Europe. Through its Work Package 5, HTx aims to assess transferability aspects of novel HTA methodology from Western Europe (WE) countries to CEE countries and give recommendations on that basis. Patient-centred and socially oriented HTA being in the focus of HTx, patient involvement in HTA was selected as a good practice to be included in such an assessment.

As the first step of our research, we mapped potential barriers to patient involvement in HTA in countries of the CEE region through a scoping literature review and discussions with CEE stakeholders (5). Based on the scientific literature, 22 potential barriers in two domains were identified. The two domains in categorizing barriers (payer/HTA body and patient) were used to simplify the survey text by noting the two different sides where these barriers typically arise. As the number of listed barriers was too high and their relative importance may not be equal for the region, we continued the research with prioritising barriers and providing recommendations only to the most relevant and important barriers. This paper describes the prioritisation process of barriers and the specific recommendations for CEE countries.

Throughout the paper – unless specified – we use the term “patients” as an umbrella term for different patient roles in representing patients. These roles were defined by European Patients’ Academy on Therapeutic Innovation (EUPATI) guidelines on patient involvement in HTA (3) as follows:

- “Individual Patients” are persons with personal experience of living with a disease. They may or may not have technical knowledge in R&D, regulatory processes or HTA, but their main role is to contribute with their subjective disease and treatment experience.
- “Carers” or “caregivers” are persons supporting individual patients such as family members as well as paid or volunteer helpers.
- “Patient Advocates” are persons who have the insight and experience in supporting a larger population of patients living with a specific disease. They may or may not be affiliated with an organisation.
- “Patient Organisation Representatives” are persons who are mandated to represent and express the collective views of a patient organisation on a specific issue or disease area.
- “Patient Experts,” in addition to disease-specific expertise, have the technical knowledge in R&D and/or regulatory affairs through training or experience, for example EUPATI Fellows who have been trained by EUPATI on the full spectrum of medicines R&D.

## Methods

**Figure 1:**
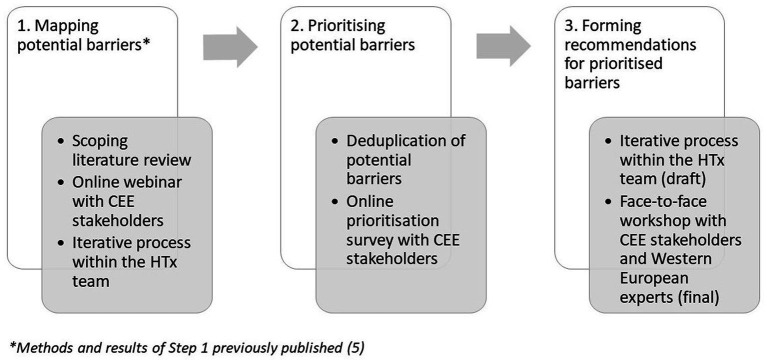
Overview of research steps.

A comprehensive overview of the different steps of the research are presented on Figure 1.

### Ranking and selection of barriers

Previous research (5) identified a total of 25 potential barriers of involving patients in HTA in CEE countries. After further deduplication of these barriers in order to prioritise the, a list of 22 potential barriers was formed: 9 barriers from the patient domain and 13 barriers from the HTA/payer domain. After creating the potential barrier list, an online ranking survey (SurveyMonkey) was developed where CEE stakeholders could score each barrier based on their priorities.

As baseline information, respondents were asked to provide their country of residence and the stakeholder group they primarily represented. Next, the survey listed all 22 barriers, and the respondent were asked to rate each barrier based on their importance using the following scale of 1 to 5:

- Very low importance (1)
- Low importance (2)
- Medium importance (3)
- High importance (4)
- Very high importance (5)

Participants of previous research steps (5) – those attending the online webinar aiming to identify potential barriers – were contacted to forward the survey to relevant stakeholders in their countries. All stakeholders were assigned to one of the following categories:

- **HTA + health policy decision maker (HPDM)**: HTA organisation representative and/or payer representative
- **Patient + caregiver**: Patient and/or informal caregiver of a patient and/or patient organisation representative and/or patient advocate and/or patient expert
- **Health care provider (HCP) + researcher:** Academic researcher and/or health care provider and/or non-academic researcher or consultant
- **Industry:** Health technology manufacturer and/or provider.

Due to the often-conflicting point of view of stakeholder groups represented in the survey, instead of a general average score, survey results were assessed by stakeholder groups. We followed two main principles:

1. Barriers receiving a high score (average score above four) by any of the stakeholder groups were automatically selected, and.
2. Stakeholder groups of patients and HTA/payer organisation representatives were designated as the most influential since their very interaction makes patient involvement in HTA possible. For that reason, the top 3 most important barriers were selected irrespective of the average score in both stakeholder groups.

### Forming recommendations

Using the principles stated above, 12 barriers out of 22 were prioritised and potential solutions were put forward. Preliminary solutions were prepared based on the literature review and iterative discussions with the two CEE partners who were both HTA experts and patient experts from the HTx H2020 consortium. The resulting draft recommendations were then reviewed and discussed by multiple stakeholders from the CEE region and HTx consortium members who were invited to a workshop, which was organized on June 2, 2022, as a satellite event of the 10th Adriatic and 7th Croatian Congress of Pharmacoeconomics and Outcomes Research in Pula, Croatia. The purpose of the workshop was to find solutions for the identified barriers with an additional input from Western European countries. Prior to the event, the proposed solutions to the barriers were sent to the invited participants to provide sufficient time to think them through. Experts from the HTx consortium were invited to help CEE stakeholders find solutions for the identified barriers with good practices from Western European countries.

1. Educational session: The workshop had an educational session, during which the following topics were discussed: (i) the perspectives of a patient organisation on patient involvement in HTA (European Organisation for Rare Diseases, EURORDIS), (ii) real-life HTA practices from the Netherlands (ZIN) and England (NICE), (iii) the importance of assessing the transferability of HTA best practices from Western to CEE countries, and (iv) and an overview of the previous steps of the research.

2. Development of recommendations: two roundtable discussions with were organized – one with patient representatives and one with HTA/payer representatives, addressing the draft recommendations. The audience was encouraged to provide feedback through live polling questions (Mentimeter) on-site and in writing after the workshop. In total, five questions were asked from the audience; two of them were single choice questions about their stakeholder group represented and country of residence and three were ranking questions. For two ranking questions on (i) preferred and most feasible method for patient involvement and (ii) most necessary skill for patients to be involved in HTA, workshop attendees were asked to pick their top 3 of the pre-defined 6 choices. Those answers ranked as first, second, and third received 3 points, 2 points, and 1 point, respectively, while those ranked fourth to sixth obtained 0 points per respondent; these points were summed up across respondents and concluded the final rank of the answers. A similar method of scoring was used for the question on the ideal profile of a patient coordinator, which had four possible answers instead of six, so respondents were asked to choose top 2 instead of top 3. Finally, there was a single-choice question with a visual scale. Workshop participants were asked which proportion of the annual HTA budget should be set to be spent on patient involvement. They were able to select a percentage between 0 and 10%. These boundaries were set up by the workshop organising team based on their expert opinion.

## Results

### Ranking and selection of barriers

A total of 105 CEE stakeholders completed the survey. Of these, 43 were representatives of HTA organisation or payer organisations, 28 were HCPs or researchers, 20 were representing patients and caregivers and 14 were affiliated with industry (Table 1). Respondents of the survey represented 13 countries and included Bulgaria, Croatia, Greece, Hungary, Kazakhstan, Lithuania, Poland, North Macedonia, Romania, Serbia, Slovakia, Turkey, and Ukraine.

**Table 1:** Number of survey respondents and workshop participants per stakeholder groups represented.

	Survey respondents	Workshop participants
HTA + HPDM	43	9
Patient + caregiver	20	12
HCP + researcher	28	13
Industry	14	5
**TOTAL**	**105**	**39**

HTA, health technology assessment; HPDM, health policy decision-maker; HCP, health care provider.

The results of the ranking survey can be found in Tables 2, 3. The top three most important barriers identified by HTA/payer organisation representatives were:

1. *No methodological guidance to support the activities of patient organisations in collecting data (*e.g.*, survey) valuable for HTA,*
2. *Fear of potential conflict of interest issues due to industry funding of patient organisations and.*
3. *Patients’ lack of experience in searching and/or interpreting information from independent resources (*i.e.*, scientific articles).*

**Table 2:** Results of the prioritisation survey per stakeholder groups I.

Barriers/Respondents			HTA + HPDM	Patient	HCP + researcher	Industry
			*n* = 43	*n* = 20	*n* = 28	*n* = 14
PAYER/HTA BODY DOMAIN	1	Limited impact of societal factors on pricing and reimbursement decisions (i.e., the reimbursement decision is evaluated only from the payer perspective per legal framework)	3.09	3.95	4.00	4.14
	2	Lack of understanding of the added value of involving patients in the HTA process	3.02	**4.10**	3.71	3.86
	3	General lack of trust in the objectivity and relevance of “patient stories” (e.g., fear of emotional aspects negatively affecting the decision-making process)	3.49	4.00	3.79	3.71
	4	Patient involvement in HTA is not mandatory/is not mentioned in the local HTA guideline	3.21	**4.05**	3.46	3.43
	5	Fear of potential conflict of interest issues due to industry funding of patient organisations	**3.88**	3.60	3.96	3.71
	6	Fear of the violation of confidentiality by patient representatives	2.81	3.35	3.21	3.43
	7	Lack of support and supporting tools (e.g., registries or network) to help patient recruitment	3.58	3.70	4.04	3.86
	8	Difficulty to identify representatives from the disease area needed (e.g., some patient communities may have “louder voices” than others)	3.23	3.70	3.54	3.50
	9	Lack of understanding of different patient roles (whether the patient is representing their own views or their patient community’s)	3.14	3.85	3.46	3.29
	10	Patient representatives might not be representative of the whole patient community in terms of socioeconomic status and other basic characteristics (e.g., higher educated, somewhat younger, health-literate patients tend to take on these roles)	3.44	3.40	3.46	3.57
	11	Payer or HTA organisations do not have enough human resources/time to involve patients (even though they would intend to)	3.42	**4.05**	3.50	3.93
	12	Lack of experience/training/skills from the HTA and payer organisations’ side in knowing how and when to incorporate patient perspectives	3.23	**4.05**	3.54	3.71
	13	Lack of local (regional or country-specific) guidelines on best practices of patient involvement to HTA	3.63	4.00	3.64	4.07

Light grey: barrier was selected due to high importance (>4) for at least one stakeholder group.

**Bold and underlined:** Top 3 of one of the two selected stakeholder groups.

HTA, health technology assessment; HPDM, health policy decision-maker; HCP, health care provider.

**Table 3:** Results of the prioritisation survey per stakeholder groups II.

Barriers/Respondents			HTA + HPDM	Patient	HCP + researcher	Industry
			*n* = 43	*n* = 20	*n* = 28	*n* = 14
PATIENT DOMAIN	14	Patient representatives’ lack of basic knowledge in HTA	3.78	3.80	4.21	3.86
	15	Patient representatives’ lack of knowledge of the local regulatory processes including how they can get involved	3.81	3.70	3.86	3.50
	16	Patient representatives’ lack of knowledge in the medical language	2.98	3.35	3.57	3.21
	17	Patient representatives do not speak/understand English which limits the amount of information (training, other countries’ experience, scientific literature) they can access	3.24	3.75	3.46	2.86
	18	No methodological guidance to support the activities of patient organisations in collecting data (e.g., survey) valuable for HTA	**4.04**	3.85	4.07	3.79
	19	Patients’ lack of experience in searching and/or interpreting information from independent resources (i.e., scientific articles)	**3.85**	3.70	3.89	3.57
	20	No fair compensation for time offered and logistics issues (e.g., traveling time and costs, documents not sent on time for review, preparatory calls or meetings during working hours)	3.47	4.00	3.57	2.79
	21	Patient organisations’ general lack of capacities due to financial constraints	3.38	**4.15**	3.71	2.86
	22	No clear rules on how to represent a patient community and how to distinguish it from representing their individual patient perspective plus confidentiality prevents patient representatives from discussing/sharing views with others before attending HTA procedures/meetings	3.83	3.30	3.68	3.29

Light grey: barrier was selected due to high importance (>4) for at least one stakeholder group.

**Bold and underlined:** Top 3 of one of the two selected stakeholder groups.

HTA, health technology assessment; HPDM, health policy decision-maker; HCP, health care provider.

The top three most critical barriers identified by patient representatives were (with a draw of three in the third place):

1. *Patient organisations’ general lack of capacities due to financial constraints,*
2. *Lack of understanding of the added value of involving patients in the HTA process,*
3. *Patient involvement in HTA is not mandatory/is not mentioned in the local HTA guideline,*
4. *Payer or HTA organisations do not have enough human resources/time to involve patients (even though they would intend to),*
5. *Lack of experience/training/skills from the HTA and payer organisations’ side in knowing how and when to incorporate patient perspectives.*

Four additional barriers were selected based on the ratings of two other stakeholder groups (Table 2). The group consisting of academic and non-academic researchers/consultants and/or health care professionals thought *patient representatives’ lack of basic knowledge in HTA*, as well as the *lack of support and supporting tools (*e.g.*, registries or network) to help patient recruitment* are critical barriers to be solved. Nonetheless, the group seemed to agree with HTA/payer representatives on the important gap of *no methodological guidance to support the activities of patient organisations in collecting data (*e.g.*, survey) valuable for HTA*. Additionally, industry representatives deemed the following barriers important: *societal factors have a limited impact on pricing and reimbursement decisions (*i.e.*, the reimbursement decision is evaluated only from the payer perspective per legal framework)* and the *lack of local (regional or country-specific) guidelines on best practices of patient involvement to HTA*.

### Workshop

The workshop had 39 participants, including 10 experts from the HTx consortium. Most participants were invited to complete the prioritisation survey prior to the workshop, except for the 6 experts from Western European countries. Workshop participants represented 9 CEE and 5 Western European countries, including Bulgaria, Croatia, France, Germany, Hungary, the Netherlands, Poland, Romania, Serbia, Slovakia, Slovenia, Spain, Ukraine, and the United Kingdom. By their main affiliation, 13 participants were HCPs/researchers, 9 represented HTA/payer organisations, 12 participants represented patients/caregivers and 5 participants represented the industry (Table 1). Of these 39 attendees, 6 participated in the roundtable of patient representatives and 12 participated in the roundtable of HTA/payer organisation representatives. Both roundtables discussed barriers arising on their behalf to be able to find implementable solutions.

**Figure 2:**
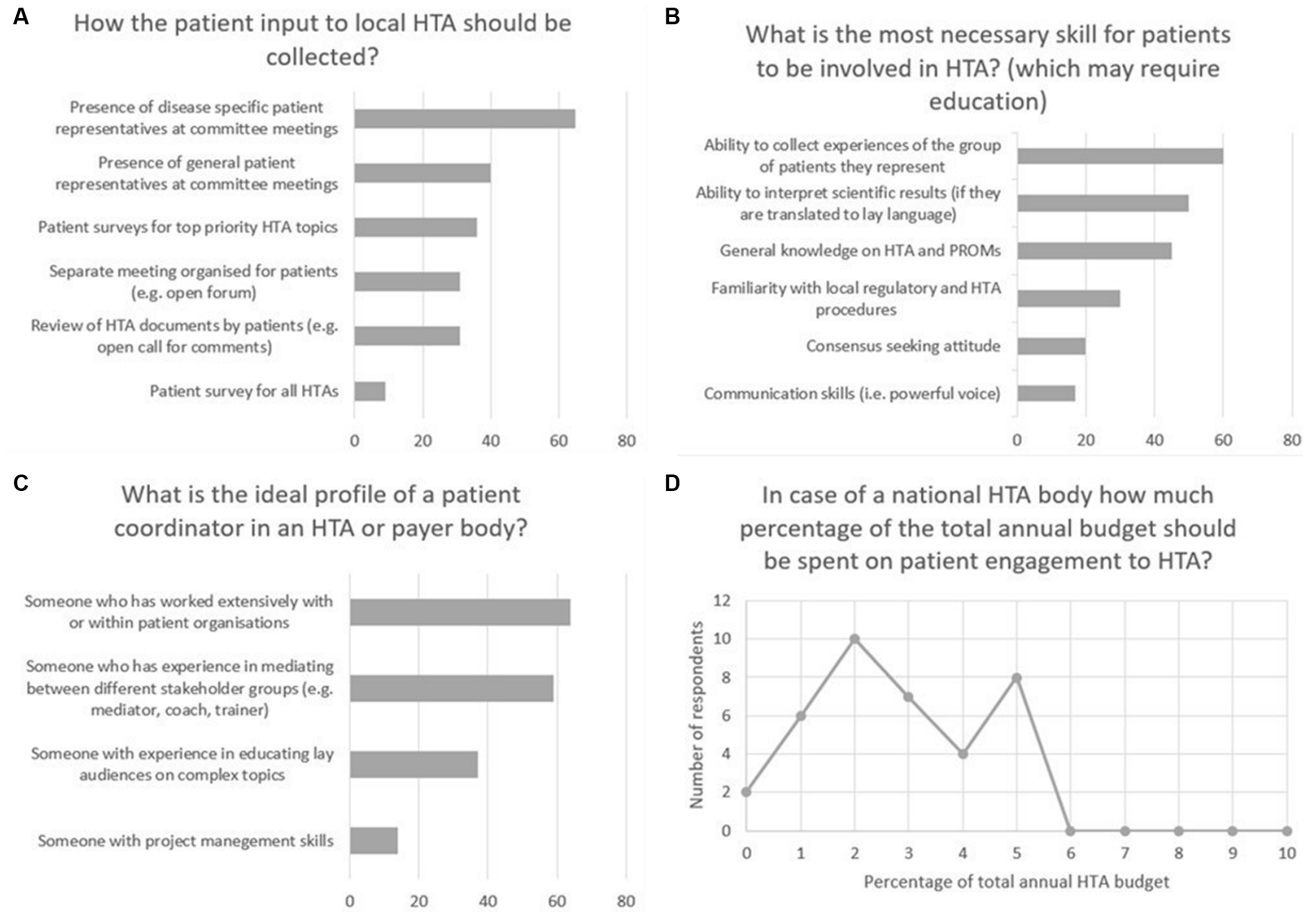
Results of the on-site voting, conducted during the workshop held on June 2, 2022, as a satellite event of the 10th Adriatic and 7th Croatian Congress of Pharmacoeconomics and Outcomes Research in Pula, Croatia. **(A)** method of patient input collection, number of respondents. **(B)** necessary skills for patients to be involved in HTA, number of respondents. **(C)** the ideal profile of a patient coordinator, number of respondents. **(D)** percentage of the total annual HTA budget to be spent on patient involvement, number of respondents, and percentage of total annual HTA budget.

According to the workshop participants, disease-specific patient representation at committee meetings was the preferred method for the collection of patient input (Figure 2A). The presence of a patient representative with general, non-disease-specific scope was marked second, while patient surveys for prioritised HTAs was marked third. Regarding the most required skills for patients to be involved in HTA, the ability to collect experiences of the group they represent was marked as first by workshop participants (Figure 2B). The ability to interpret scientific results and general knowledge on HTA and PROMs were voted on as second and third, respectively. On the question of the ideal profile of a patient coordinator in an HTA or payer organisation working experience with or within patient organisations was marked as most important (Figure 2C). Mediation skills were voted as second and experience in educating lay audiences on complex topics was marked as third. Workshop participants were asked which proportion of this annual budget should be set to be spent on patient involvement. The most voted options were 2, 5 and 3%, in descending order, whereas the weighted average of all answers was 2.78% (Figure 2D).

### Recommendations

Recommendations resulting from our multi-step research are presented below. Related barriers are referenced for each recommendation in Table 4, per the barriers’ number as seen in Tables 2, 3.

**Table 4:** Recommendations for involving patients into HTA in CEE countries.

Recommendations	Barriers
#1 Educate HTA/payer organisations on the value and good practices of patient involvement.	2, 12
#2 Acknowledge patients as experts on their condition similar to health care professionals. Differentiate but equally value the input of individual patients, patient representatives and accredited patient experts.	2
#3 Revise local HTA guidelines and procedures: Step 1: Involve patients into the revision Step 2: Revise how patients are involved in the HTA and decision-making process from the beginning to the end. Use and adapt a mix of existing tools and practices for patient involvement in HTA, implemented gradually. Step 3: Revise how societal factors, the patient experience and perspectives are considered in the evaluation framework of health technologies Step 4: Regularly monitor whether the guidelines are followed and prepare impact assessment.	1, 4, 7, 12, 13
#4 Nominate a dedicated person/team to be responsible for patient involvement activities with sufficient available capacities at each relevant HTA and decision-making body.	11, 12
#5 Set a certain percentage of the public HTA annual budget to be spent on patient involvement as a goal.	11
#6 Fair compensation for time and transportation should be provided for the patients involved in the HTA process.	21
#7 EU-funded calls for the implementation of patient-centric evaluation of health technologies especially in countries with limited experience in patient involvement.	11, 12, 13
#8 Set up an open call for individual patients or patient organisations to register for involvement into HTA. Have and implement a clear policy on conflict of interests.	5, 7
#9 Provide tailored training(s) and training materials for patients on HTA and local health policy decision-making procedures. Set up a working group of organisations with extensive experience in education and working with patients to act as centre of training of patient experts.	14, 18, 19
#10 Education of patient organisations on collecting data and interpreting scientific evidence based on international educational resources.	18, 19
#11 Patient organisations to aim for a diversified portfolio of funders.	5
#12 Normative state funding for NGOs with close auditing and detailed expectations from and responsibilities of patient organisations. Neither public, nor private funding should be banned by legislation.	21

#### #1 Educate HTA/payer organisations on the value and good practices of patient involvement

It is essential to educate HTA/payer organisations on the value and good practices of patient involvement. Education on this matter should come from reliable and acclaimed sources. International umbrella patient organisations, academics with research in the field or other countries’ HTA organisations with long-time experience in patient involvement can help demonstrate its value and good practices. However, it is also important to include local patients, patient representatives and patient experts in the educational efforts to help start a conversation between the different stakeholders and showcase the valuable work they are doing locally.

#### #2 Acknowledge patients as experts on their condition, similar to health care professionals. Differentiate but equally value the input of individual patients, patient representatives, and accredited patient experts

All parties should acknowledge the irreplaceable value of the patients’ voice in health care decision making. As a rule of thumb, where HCPs can be involved in the HTA and reimbursement decision-making process as experts, patients can and should be involved as well.

It is also essential to clearly differentiate the potential roles of patient interaction such as individual patients, carers, patient advocates, patient organisation representatives, and patient experts (3). HTA/payer organisations should choose the most adequate patient representation for each activity (3). It could be considered to involve patients from all these roles simultaneously as they bring different perspectives to the table, with equal credibility as other experts.

#### #3 Revise local HTA guidelines and procedures

We suggest the creation/revision of local HTA guidelines (methodological guidelines for establishing relative effectiveness and economic evaluations), operating procedures and decision-making procedures using the following steps:

**Step 1**: Involve patients into the creation/revision.

It is advised to involve patients and the public into the creation/revision and make it a collaborative, transparent process. If patients are not involved, guidelines or processes cannot be truly patient-centred. Patients should have at least a commenting right with support provided to understand the details of the current/forming guidelines and procedures.

**Step 2**: Revise how patients are involved in the HTA and decision-making process from the beginning to the end. Use and adapt a mix of existing tools and practices for patient involvement in HTA, implemented gradually.

We recommend a mix of patient involvement methods, with strong emphasis on involving disease-specific patient representatives at committee meetings (Figure 2A). Involving general patient representatives at committee meetings and conducting surveys for the greater patient community in prioritised cases of HTA are desired methods for patient involvement as well. The review of HTA documents by patients (with a reasonable deadline and lay language support); and separate meetings organized for patients only can also help in collecting patient input at certain points of the process.

Systematically revise all steps of the HTA and decision-making process with patients to find the best ways to involve patients at given points of the process. With long-standing international experience available, the revision should rely on the learnings of good practices, adapted to the local context.

Another key aspect is the time needed: HTA/payer organisations need to be aware of the time it takes to include patient input, and timelines of their procedures should be adopted accordingly.

**Step 3**: Revise how societal factors, the patient experience and perspectives are considered in the evaluation framework of health technologies.

It is not enough to revise how patients are involved in the HTA process, the revision should also cover which societal factors and patient experience elements are considered for the decision and how. This will also help to create space for channelling and considering the patient input provided throughout the HTA processes (6). Such societal factors and patient experience elements could include improved access for vulnerable patient populations, responsiveness to the patient’s individual needs (e.g., convenience of treatment administration), caregiver quality of life, or the household’s financial burden (6).

**Step 4**: Regularly monitor whether the guidelines are followed and prepare impact assessment.

To make sure guidelines are followed, follow-up and regular impact assessments are needed. If pre-defined goals are not met, the process should be iterated, and additional measures should be taken. Making patient involvement mandatory instead of recommended might be considered in certain legislation frameworks.

The quality of the interaction between patients and HTA/payer representatives should be monitored post-input (e.g., by questionnaires), both for patients and for HTA/payer representatives to explore whether their respective expectations were met, whether patients feel their opinions were listened to, whether the process was informed by the patient input, as a basis for impact assessment and further improvement of the procedure. Conclusions should be followed-up on with patients who have been involved.

#### #4 Nominate a dedicated person/team to be responsible for patient involvement activities with sufficient available capacities at each relevant HTA and decision-making body

A dedicated person, ideally a dedicated team should be responsible to coordinate patient involvement activities within the HTA/payer organisation. The most influential skills to look for when choosing a person or building a patient coordinator team include (1) having worked with or within patient organisations before, (2) having experience in mediation between different stakeholders, and (3) having experience in translating complex topics to lay language (Figure 2C).

#### #5 Set a certain percentage of the HTA annual budget to be spent on patient involvement as a goal

To have sufficient human resources to conduct patient involvement activities meaningfully, HTA/payer organisations need dedicated funding. Our recommendation is to set a certain and meaningful percentage of the public HTA annual budget to be spent on patient involvement as a goal (Figure 2D). However, what is deemed as the “HTA annual budget” can be defined in various ways across different countries, thereby the target percentage can change country-by-country.

#### #6 Fair compensation for time and transportation should be provided for the patients involved in the HTA process

Fair compensation for time (i.e., lost revenues) and covering transportation costs should be the base principle when involving patients in the HTA process. This is the bare minimum to allow patients to be able to participate in such processes (e.g., especially for those living in the countryside and/or experiencing financial hardship).

#### #7 EU-funded calls for the implementation of patient-centric evaluation of health technologies especially in countries with limited experience in patient involvement

Another way of gaining additional funding for the implementation of patient involvement practices is to invite applications through EU-funded calls. We propose designing a call targeting the advancement of countries with limited experience in patient involvement (mostly but not exclusively CEE countries), in which they can apply for EU-funding for educational and capacity building activities or specific case studies.

#### #8 Set up an open call for individual patients or patient organisations to register for involvement into HTA. Have and implement a clear policy on conflict of interests

Patient involvement should be open to all and non-discriminative on the grounds of previous experience and presumed support time needed. There are multiple initiatives locally and internationally aiming to ease patient recruitment with education, coordination and/or databases. We recommend organisations leading these initiatives to come together and join forces on the base of commonly agreed principles. We also recommend local HTA/payer organisations to set up their open call for local patients, carers, patient advocates, patient experts and patient organisation representatives to be able to express interest. We suggest to actively promote this opportunity to harder to reach patient communities and a periodical revision of the registry.

Regarding ethical and compliance issues, a clear policy on financial and other conflicts of interests (how interests are declared, assessed and addressed) should be in place. Those registered should complete a declaration of interest form both on personal and organisational level conflict of interests and update it periodically. It should be clarified what kind of involvement with industry (e.g., attending a single advisory board meeting with a company versus only in case of direct conflict of interest) would impose restrictions on how a person can be involved in the HTA and decision-making process. We argue that in situations where patient experts and/or patient organisation representatives are difficult to identify, a softer approach should be taken and special measures could be proposed, equivalent to the “expert witness” status at the European Medicines Agency (EMA). Expert witnesses can be heard or participate in the deliberations but are not allowed to take part in the vote. However, the consequences of not being transparent with potential conflict of interests should be serious and communicated clearly from the beginning.

#### #9 Provide tailored training(s) and training materials for patients on HTA and local health policy decision-making procedures. Set up a working group of organisations with extensive experience in education and working with patients to act as centre of training of patient experts

Patients’ lack of knowledge in HTA has been marked as a main barrier by HCPs and researchers. It is essential for patients to understand the need for HTA and rationale behind it. Patient organisations working on an international level, and academics have a main role in education on these aspects as well. Additionally, as health policy decision-making processes differ from country to country (in some cases even by regions/municipalities of a country), it is important for patients to understand the local process they want to give their input to. Local HTA/payer organisations and scientific societies in the field of health economics (e.g., ISPOR local chapters) should play a leading role in providing and organising such educational activities and materials.

Regarding the patient experts who can act as representatives of the general patient community and mediators, we recommend that a training centre should be set up locally, adapting existing training materials (e.g., EUPATI) and utilising local organisations’ experience and infrastructure in education and/or working with patients (e.g., non-governmental organisations (NGOs), academia). Ideally, the initiative should be bottom-up but coordinated centrally.

#### #10 Educate patient organisations on collecting data and interpreting scientific evidence based on international educational resources

We recommend educating patient organisation representatives and patient experts on conducting their own research on patient input collection and interpreting scientific evidence. Available training materials and good practice documents should be translated and used as educational resources.

#### #11 Patient organisations to aim for a diversified portfolio of funders. And to declare funding sources publicly

Patient organisations should aim for a diversified portfolio of funders. Avoiding a single funder to be proportionally standing out of the funding scheme can be a piece of guarantee for independency. Ideally, funding diversity should be obtained both in terms of public-private mix and within these categories, i.e., at least 3 different private organisation funders. However, it is important to mention that an ideal mix is rarely obtainable, for example in countries with limited or no public funding or in disease areas with limited or no treatment options.

#### #12 Normative state funding for NGOs with close auditing and detailed expectations from and responsibilities of patient organisations. Neither public, nor private funding should be banned by legislation

Our main recommendation to overcome patient organisations’ general lack of capacities due to financial constraints is to provide them normative state funding. Criteria and auditing of such funding should be strict to avoid misuse of the funding. Some countries when introducing normative state finding also introduced a ban on private funding for patient organisations, however, funding from private sources is also essential to obtain the ideal mix of funding. This way patient organisations can have an independent and critical voice with both public and private organisations without the fear of bankruptcy. Thereby, neither public, nor private funding should be banned by legislation.

## Discussion

In this paper we present the ranking of previously identified barriers to meaningful, systematic, and sustainable patient involvement in HTA in CEE countries, as well as the development and the resulting 12 recommendations aiming to answer these barriers.

### Ranking results of different stakeholders

Technically, there was no overlap between the main barriers prioritised by HTA/payers, and patients. HTA/payers seem to prioritise their need for evidence-based and unbiased patient representation. In the meantime, patients perceived the general lack of funding and capacities of patient organisations as the greatest hurdle. However, patients’ experience of lack of funding mirrors HTA agencies’ and payer organisations’ fear of patient organisations being funded and influences by industry. The other four barriers prioritised by patients highlighted the perceived shortcomings of HTA/payer organisations and policies in involving patients to HTA, such as – from the participating patients’ perspective –not understanding the value patient can bring to the process, not having enough resources, skills, and training to involve patients, and that patient involvement not mentioned/mandatory by the local HTA guideline. As for other important barriers, HCPs and researchers added two barriers to the list, a third one being an overlap with HTA and payer organisation representatives, while industry representatives added two barriers with no overlap with any other stakeholder group.

### Forming recommendations

The face-to-face workshop in Pula, Croatia included two roundtable discussions with live polling questions to the audience. Live polling had proven to be fruitful way of discussing recommendations and providing a feasibility check to experts in the room.

Two top rated barriers from the patients’ perspective were the perceived *HTA/payer personnel’s lack of understanding of the added value of involving patients in the HTA process* and their *lack of experience/training/skills in knowing how and when to incorporate patient perspectives*. Workshop participants agreed that educating HTA/payer organisations on these matters, sharing good practices and examples of the impact of patient input on the quality of the assessment would be a key step towards making a difference. Workshop participants pointed out that, just as health care providers (HCPs), patients could provide hands-on perspectives of the specific disease, in which the two types of perspectives would be complementing, but not substituting each other. The main conclusion was that in all steps where HCPs input would be expected, patients should be involved as well.

*HTA/payer organisations not having sufficient human resources to involve patients* was marked as an important barrier by patients in the survey. Additionally, workshop participants highlighted the importance of accumulated experience with patient involvement reflecting a natural learning curve. Workshop participants mentioned how important it would be to transform these learnings from personal into organisational knowledge, as often with the fluctuation of employees, good initiatives and the knowledge gained would disappear from the organisation. Also, patient organisations are prone to member attrition, especially in more severe illnesses and conditions. A good example is the National Institute for Health and Care Excellence (NICE), where a whole team of experts is responsible for patient engagement assuring adequate capacities and continued practices.

When discussing funding for patient involvement and setting a certain percentage of the total HTA annual budget of the country, some workshop participants questioned whether there would be such a budget made available in all CEE countries. It was argued in the workshop that all countries in which manufacturers/technology providers were expected to submit an evaluation of the health technology for review before reimbursement should have such an annual budget hence a certain percentage of it can be targeted as a performance indicator.

The lack of support and supporting tools to help patient recruitment was marked as an important barrier to patient involvement by HCPs and researchers. As mentioned before, there are multiple initiatives (locally and internationally) aiming to ease patient recruitment, but as workshop attendees commented, these would be often segmented and not offering a proper solution. There was a debate on the need of centralization versus decentralization on the European versus local levels when setting up initiatives like individual patients or patient organisations registering for HTA involvement. Centralised initiatives on the European level might help to avoid duplication of work and be more effective, however, the difference in legal, procedural and financial aspects of these countries and language barriers might limit the broadness of reach especially in CEE countries.

Workshop attendees added the need to involve other than the “loudest” voices of large patient organisations, who were well-known by decision-makers. They pointed out that smaller patient organisations were often overlooked and not invited to the conversation, even if they expressed their interest to be involved. They also proposed the model of the European Medicines Agency (EMA).^4^ EMA allows that anyone can express interest to get involved in EMA’s work on their website, and they are contacted when a relevant submission is under evaluation; those registered need to affirm their continued interest periodically to stay in the database.

### Implications of results

Value-based care cannot be implemented without the engagement of patients in the most important health policy decisions. Our research has confirmed that short-term *ad-hoc* interventions may not improve the patient engagement into HTA in CEE countries, specific actions are needed based on long-term policy objectives and with strong political support. There are two main target groups of the recommendations: (1) patients, particularly patient organisation representatives and patient experts, and (2) representatives of HTA organisations, payers, and health policy decision-makers. The appreciation and mutual recognition of added value of these two stakeholder groups is essential for the successful implementation of the recommendations. Other stakeholders, such as health care professionals, academic and non-academic researchers and consultants play a significant role as facilitators and educators in the process. Some of our recommendations target an international audience, like decision-makers from the EU-level in case of our recommendation for EU-funded calls. Additionally, many of our recommendations can be relevant for non-CEE countries. We urge local stakeholders to use our recommendations as an educational and advocacy tool if needed and aim to start a conversation with all stakeholders at the table.

There are a few expected changes concerning the new EU-wide HTA regulations,^5^ even for CEE countries that are not yet EU members. An important takeaway is that local HTA will be still needed after the final framework for join HTA has been established, therefore efforts are still required on the country level to improve patient involvement.

On the other hand, joint HTA opens the possibility for centralised patient involvement in assessing the relative effectiveness of a health technology. However, due to language barriers and capacity limitations of CEE patients/patient organisations, it might be more challenging to involve CEE representatives. Therefore, extra efforts may be needed from the centralised HTA organisation to reach equal geographical representation. Overall, we believe that our recommendations are still going to be needed and valid, after the new regulation sets in place.

Prior to HTA, industry and academia have an essential role in involving patients from the earliest phases of the research and development process of new health technologies (7, 8). Moreover, generating data on patient-relevant outcomes such as quality of life, patient preferences, and patient experience is critical in providing evidence on the value for patients (9–11). Advancing and using these measures is the responsibility of industry, however, they should be incentivised by relevant HTA and payer bodies to do so (11).

### Available resources

To support the implementation of our recommendation, the following section lists useful international resources aiding different phases of execution from advocacy through education, forming the patient involvement process to post-input impact assessment:

- The European Patients Academy on Therapeutic Innovation (EUPATI) published a guideline on patient involvement in HTA, with suggested working practices relevant describing concrete steps to building a system with patients involved (3).
- A Joint Statement of 14 European patient organisations was positioned in 2018, calling for meaningful patient involvement in European cooperation on HTA, which served and still serves as a successful advocacy tool.^6^
- The European Network for Health Technology Assessment (EUnetHTA) has a guidance document on collecting patient input in their relative effectiveness assessments.^7^
- The European Patients’ Forum (EPF) report on the added value of patient organisations^8^ might help in advocating for the needs of patient organisations in terms acknowledgement and funding.
- Results of the IMI-PARADIGM (2018–2020) project offer a unique framework for patient engagement practices including impact measures, remuneration and sustainability domains (12, 13).
- Results of the Erasmus+ Values in doing assessments of health technologies (VALIDATE) project,^9^ suggesting a concrete methodology that can help HTA practitioners to integrate empirical analysis and normative inquiry in a transparent way, hence offering a distinct way of giving stakeholders a structural and constructive role in HTA (14).
- Health Technology Assessment International (HTAi) has an interest group with various available resources and frequent publications called the Patient and Citizen Involvement Interest Group (PCIG). The HTAi PCIG has plain language summaries of HTA, written patient input submission templates for different kinds of health technologies (medicine, non-medicine, diagnostic) in a growing number of languages, as well as guides on collecting patient-based evidence and many more on their website.^10^
- Numerous HTA bodies publish guides and materials aiding patient involvement like the National Institute for Health and Care Excellence (England),^11^ the Scottish Medicines Consortium (Scotland)^12^ and Canada’s Drug and Health Technology Agency (Canada).^13^ The Patient Involvement Interest Group of the Spanish Network for Health Technology Assessment of the National Health System (RedETS) developed and published a decisional flowchart for meaningful patient involvement in HTA (15).

## Limitations

Our study has some obvious limitations. Countries in the CEE region are diverse in many ways, including economic measures, health care budget, development level and process of HTA, as well as health policy decision-making. Given this heterogeneity, we did not attempt to make specific recommendations for each CEE jurisdiction, but prepared general recommendations for the entire CEE region, which were based on the barriers prioritised in the ranking survey. Additionally, there were some CEE countries where we could not involve any stakeholders from, and those involved were not balanced in terms of countries represented. A main limitation of the broadness of reach of the study was the language barrier. The language of the survey, as well as the language of the workshop were English, thereby those without a working knowledge of English could not participate. Throughout the face-to-face workshop, close-ended questions were used for the interactive poll (mentimeter), allowing participants to select only from a limited number of options pre-determined by workshop organisers.

## Conclusion

In this paper we present 12 recommendations for meaningful, systematic, and sustainable patient involvement in HTA in CEE countries, considering the identified major barriers. We urge local stakeholders to use our recommendations as an educational and advocacy tool if needed and aim to start a conversation with all stakeholders at the table. Our hope is that engaging more than a hundred CEE stakeholders during the study helped to spread awareness on the importance of patient involvement and the resulting recommendations provide tangible steps for the way forward. Future studies shall focus on country-specific case studies of implementing recommendations.

## Data availability statement

The raw data supporting the conclusions of this article will be made available by the authors, without undue reservation.

## Ethics statement

Ethical review and approval was not required for the study on human participants in accordance with the local legislation and institutional requirements. Written informed consent for participation was not required for this study in accordance with the national legislation and the institutional requirements.

## Author contributions

ZK, BN, IJ, ZP, and MD: conception and design. IJ, ZP, and BN: analysis and interpretation of data. IJ and MD: drafting of the manuscript. FH, TB, MF, ZMa, IB, CA, KrT, OP, RH, IG-I, MC, TT, MN, LL, MK, MM, AS, ZMi, KoT, DaD, DiD, SK, and WG: critical revision of the manuscript for important intellectual content. WG and ZK: supervision. All authors contributed to the article and approved the submitted version.

## Funding

The HTx project has received funding from the European Union’s Horizon 2020 research and innovation program under grant agreement N° 825162. This dissemination reflects only the author’s view and the Commission is not responsible for any use that may be made of the information it contains.

## Conflict of interest

KrT, RH, ZP, BN, and ZK were employed by Syreon Research Institute. At the time of the study IJ was the President of the European Patients’ Forum Youth Group, a Board of Trustees member at the EUPATI Foundation and employed by Syreon Research Institute. MN is the founder and co-owner of HealthQuest, a health technology assessment and market access consulting company. DaD is a Trustee of Thrombosis UK. TB was employed by Patvocates GmbH.

The remaining authors declare that the research was conducted in the absence of any commercial or financial relationships that could be construed as a potential conflict of interest.

## Publisher’s note

All claims expressed in this article are solely those of the authors and do not necessarily represent those of their affiliated organizations, or those of the publisher, the editors and the reviewers. Any product that may be evaluated in this article, or claim that may be made by its manufacturer, is not guaranteed or endorsed by the publisher.

## Footnotes

^1^https://www.eunethta.eu/

^2^https://eur-lex.europa.eu/legal-content/EN/TXT/?uri=CELEX:32021R2282

^3^www.htx-h2020.eu

^4^https://www.ema.europa.eu/en/documents/other/engagement-framework-european-medicines-agency-patients-consumers-their-organisations_en.pdf

^5^https://health.ec.europa.eu/health-technology-assessment/regulation-health-technology-assessment_en

^6^https://www.eu-patient.eu/news/latest-epf-news/2018/patient-organisations-have-co-signed-a-joint-statement-on-hta/

^7^https://www.eunethta.eu/wp-content/uploads/2019/06/Final_290519_Patient-Input-in-REAs.pdf?x69613

^8^https://www.eu-patient.eu/globalassets/library/publications/epf_added_value_report_final.pdf

^9^www.validatehta.eu

^10^https://htai.org/interest-groups/pcig/resources/

^11^https://www.nice.org.uk/about/nice-communities/nice-and-the-public/public-involvement/public-involvement-programme/patient-public-involvement-policy

^12^https://www.scottishmedicines.org.uk/about-us/public-involvement/

^13^https://www.cadth.ca/patient-and-community-engagement

## Abbreviations

CEE: Central and Eastern European
EMA: European Medicines’ Agency
HCP: Health care provider
HPDM: Health policy decision-maker
HTA: Health technology assessment
